# Women’s Views on Handling and Managing Their Breast Cancer in Pakistan: A Qualitative Study

**DOI:** 10.3390/diseases4020017

**Published:** 2016-04-14

**Authors:** Najma Naz, Sabiha Khanum, Grace Teresinha Marcon Dal Sasso, Maria de Lourdes de Souza

**Affiliations:** 1 Postgraduate Program in Nursing (GIATE/UFSC), Federal University of Santa Catarina, Florianópolis/SC 88040-900, Brazil; grace.sasso@ufsc.br; 2 Postgraduate Program in Nursing (PEN/UFSC), Repensul Institute, Federal University of Santa Catarina, Florianópolis/SC 88040-900, Brazil; sabiha.khanum@hotmail.com (S.K.); repensul@uol.com.br (M.L.S.)

**Keywords:** cancer, breast cancer, Pakistan

## Abstract

In this study, we examine and analyze the experiences of women and their perceptions on handling and managing their breast cancer. Seven women from Peshawar, Pakistan who had breast cancer and have been cured, were interviewed. Qualitative content analysis of their life stories was performed using a computerized software Atlas.ti. In the coding process, 128 codes were initially developed. These codes were then grouped into 12 categories, which were then further refined into 8 theoretically grounded categories: awareness and education about breast cancer, cultural barriers, early detection, quality of care and treatment, support, side effects, courage and learned to face challenges. The early views of participant’s feelings about breast cancer are mostly similar to the general population in Pakistan. Before starting treatment, all participant were unaware of the treatment process and had fear in their mind. They were hesitant in starting their treatment and were worried. However, when they were cured, their attitudes toward breast cancer and even to their whole lives were changed. Comprehensive awareness programs in a culturally acceptable language and facilities for routine breast examinations should be easily accessible to all women in Pakistan in order to promote early detection. In order to eradicate cultural barriers, female staff who are trained to perform routine breast examinations, should be available in all facilities and treatment centers.

## 1. Introduction

Breast cancer, frequently diagnosed in women, is due to the presence of malignant tumor in breast tissues. Cancer cells continue to grow with time and form new abnormal cells, instead of dying. Cancerous cells can also invade or spread into nearby tissues. Growing out of control and invading other tissues are what makes a cell a cancer cell.

The incidence of breast cancer varies worldwide, ranging from 3.9/100,000 in Mozambique to 101/100,000 in the U.S [1,2]. It is the most frequently diagnosed cancer in women both in the developed and developing countries [3]. An estimated 508,000 women died in 2011 alone due to breast cancer worldwide [4]. Breast cancer is the most frequently diagnosed cancer in females in the United States, affecting 1 in 8 women [4]. It is believed to be a disease of the developed countries, but almost 50% of breast cancer cases and 58% of deaths occur in less developed countries [5].

Survival rates from breast cancer also vary worldwide, ranging from 80% in North America and other developed countries to about 60% in middle-income countries and below 40% in low-income countries [6]. Due to the lack of education, awareness, early detection schemes, adequate diagnosis and treatment facilities, the rate of survival is low in less developed countries.

**Figure 1:**
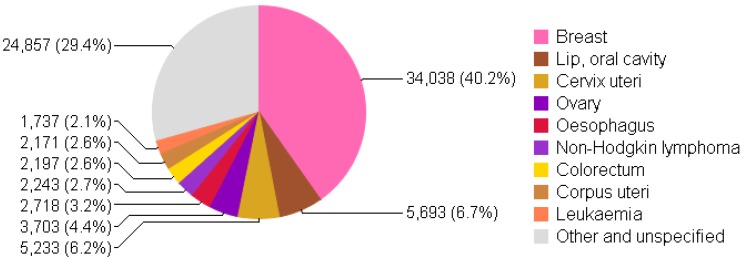
Incidence of cancer in females of all ages in Pakistan (per 100,000) (chart adapted from [10]).

**Figure 2:**
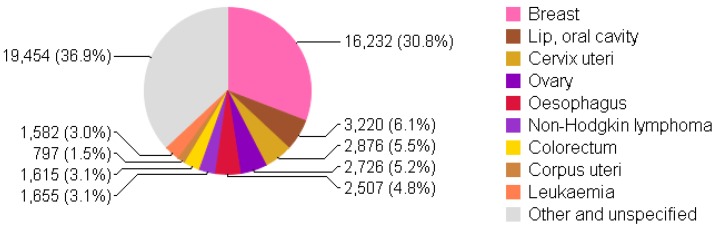
Mortality due to cancer in females of all ages in Pakistan (per 100,000) (chart adapted from [10]).

Pakistan has the highest incidence rate of breast cancer in Asia, where one out of every nine women develops breast cancer [7]. Figure 1 and Figure 2 shows the incidence and mortality due to breast cancer to be on top in females of all ages in Pakistan in 2012. A recent report from Shaukat Khanum Memorial Cancer Hospital and Research Center (SKMCH & RC) in Lahore, Pakistan shows 45.9% of malignancies to be breast cancer among adult females from December 1995 to December 2009 [8]. Breast cancer statistics in Pakistan have not been exact or adequate due to the absence of a cancer registration system at national level [8,9].

Screening and early detection of breast cancer are poor in Pakistan. Note that, at SKMCH & RC (the leading cancer treatment center in Pakistan), more than 30% of the breast cancer is diagnosed in stages III and IV [8]. Early detection of breast cancer by mammography is not possible in a country with low resources and a high population such as Pakistan. The amount of financial resources, infrastructure and health professionals are not sufficient to handle mass screening in our target population. Due to the lack of awareness about breast cancer, women in Pakistan only go to health professionals when their cancer has reached an advanced stage.

Breast self examination (BSE), clinical breast examination (CBE) and mammography are the commonly recommended screening methods for breast cancer [11]. BSE is a check-up that women can do at home by looking and feeling each of the breasts for changes. Development of a mass in the breast that feels different from the rest of the breast tissue is the first observable symptom of breast cancer. Other signs may include thickening of breast tissue, the difference in sizes of breasts, rashes around the nipple, discharge from the nipple, continuous pain or swelling in the breast or armpit [12]. BSE is an easy, inexpensive and simple procedure which does not require professional expertise and can be carried out by women themselves. Regular BSE can increase the chances of early detection which ultimately results in high survival rate.

The diagnosis mechanism of breast cancer includes a physical examination, mammogram, breast ultrasound, MRI, biopsy, and ductogram. Breast cancer is usually treated with surgery, hormone therapy/chemotherapy/radiation therapy, or a combination of these and chances of survival typically depends on the stage at which it is diagnosed [13].

Due to increased screening, earlier detection programs and improved treatment for breast cancer, the death rate from breast cancer has declined to about 20 percent over the past decade [14]. One of the main focusses in fighting breast cancer has been to promote early detection. With early screening, the disease can be detected at an earlier stage which results in a higher chance of successful treatment. In the case of early detection, the affected tissue can be removed before it spreads to other areas of the body and the chance of survival is increased.

It is important to minimize delays in detection, diagnosis, and treatment because the later the stage of breast cancer, the more the complications will be. There are two major types of delay in breast cancer [15].

- **Patient delay** which is the delay in seeking proper medical treatment after discovering a potential breast cancer symptom.
- **System delay** which is the delay within the health care system in getting appointments, diagnostic tests, and initiating treatment.

Women in developed countries did not perceive breast cancer as a biggest threat to their health because of improved breast health awareness. In a study [16] conducted in USA, with 41 participants including white, black, Hispanic, Asian, and Native Americans, only one-quarter of women felt that breast cancer is a fatal disease. Forty participants in this study knew the importance of breast health awareness and early detection, paraphrasing the idea that “early detection saves lives”.

Asian women have limited breast health awareness due to cultural barriers, religion, personal modesty, and reluctance to visit a male doctor [17]. In a study [18] conducted in Pakistan and the UK, on the views of 44 Muslim women about breast health practices, the authors found that the lack of breast health awareness, modesty and taboos related to breasts, and lack of national breast cancer screening program were major hurdles in increasing survival rates from breast cancer in Pakistan.

The purpose of this study is to understand women’s perceptions, experiences, and knowledge in handling and managing their breast cancer from diagnosis to recovery. These views and experiences will serve as guidelines and a source of motivation for breast cancer patients and will help clinicians, caregivers, and family members to effectively control and manage this curable disease in Pakistan.

## 2. Methodology

Purposive sampling was used to select women with breast cancer experience from Peshawar, Pakistan. Women aged between 20 and 60 years who belonged to the socio-cultural profile of Pakistan and who had breast cancer and had been treated and cured were the inclusion criteria for this study. Women were identified through private and public cancer research centers and hospitals in KPK, Pakistan. Potential target women were approached and written consents were obtained for participation in the study. Participants were given written information in Urdu outlining the ethics of participation assuring anonymity, confidentiality, and voluntary participation.

This study consists of life stories of seven women, aged between 25 to 60 years, who had breast cancer and have been treated and cured. All women were asked one question, to tell their complete story from breast cancer detection to treatment and successful recovery. Additional focused questions were asked (in a second session) if the authors felt that some of the relevant aspects are not addressed. For instance, “what was your reaction when you heard about your breast cancer?” or “What really helped you during your treatment of breast cancer?”. All interviews (each one took two sessions) were held in a local community health center between 1 June 2015 and 15 October 2015 and took approximately one hour each. A second session with each participant was arranged in order to verify that all information obtained was accurate and complete. The life stories were digitally recorded and transcribed verbatim. Transcribed written stories about each woman were read and verified and the correctness of transcripts made from audio recordings was checked independently by first two authors. Content analysis was then performed and the material was coded in an English version of ATLAS.ti version 7.1.8 (a software package that provides systematic procedures and support in the analysis of qualitative data research developed by Scientific Software Development GmbH, Berlin, Germany).

The life stories of all the women were analyzed using established methods for qualitative content analysis [19]. The coding process was performed using ATLAS.ti, during which codes were made. Codes were then refined and grouped into relevant categories so that it could then be described qualitatively [20].

Transcripts analysis and interpretations were based on coding instructions [20] in order to be in agreement with qualitative research [21]. In this way, theoretically grounded and measurable categories were obtained which were then used to substantiate the valid arguments regarding the perceptions and views of women on handling and managing their breast cancer.

## 3. Results and Discussion

The sample of women interviewed was small yet fairly diverse in terms of marital status, age, education, occupation and economic conditions. The sample comprised four married (with one to six children), one widow (with three children) and two single women. Their ages were 25, 29, 33, 42, 37, 56 and 60 years. Their education ranged from 10th grade to master degree (two women with 10th grade, one 12th grade, three graduation and one master). Of these, four women were housewives, one student, one school teacher, and one bank employee. One woman belonged to lower class, three to lower-middle class, three to upper-middle class (judged approximately). Only one woman had a history of breast cancer in her first-degree female relative. The economic conditions of three women were good and affordability of breast cancer treatment was not a problem for them. Four women completed their treatment in SKMCH & RC, a cancer specialized hospital located in the city of Lahore and Peshawar in Pakistan, where poor and needy patients are treated free of cost or at subsidized rates, according to their economic conditions.

The first and most common operations when working on qualitative data is the marking of segments of data and assigning codes to them. In the coding process, 128 codes were initially developed. These codes were then grouped into 12 categories. These 12 categories were then further refined into 8 theoretically grounded categories: awareness and education about breast cancer, cultural barriers, early detection, quality of care and treatment, support, side effects, courage and learned to face challenges.

Differences between the participants (such as age, occupation and the environment from which they came, *etc.*) did not affect much the experiences and views of women before starting treatment and after they were cured. The views and experiences of these women in the form of eight emerged themes are further discussed below.

### 3.1. Awareness and Education about Breast Cancer

The level of knowledge regarding breast cancer varied among participants. Three out of the seven women knew nothing about breast cancer before their diagnosis. Two of them had some knowledge about this disease. Two of them knew about breast cancer and its symptoms from their friends, women’s magazines and the internet. Interest in one of the two women who knew about breast cancer, developed when she saw a news on internet that:

P 6: “*In Pakistan, breast cancer is the most common cancer in females*”

One of the women expresses that:

P 2: “*I always thought that only older women can have breast cancer*”.

Five women did not know about breast cancer screening and were not sure where to go and who to contact about screening. Only two women had some knowledge about different factors associated with breast cancer such as screening, side effects, and treatment. No-one in the participants knew anything about the etiology of breast cancer.

There were practically no differences in the views of interviewed women with regard to the fear of breast cancer at the initial stage. Six of the women thought that breast cancer is a fatal disease while one thought that it is a dangerous one. This misconception was due to the lack of awareness and no forum to openly discuss this disease. The initial fear and misunderstandings of women about breast cancer were then later changed when they started their treatment and knew more about the disease. In developing and poor countries, most of the women do not know about screening and cancer treatment and hence their cancer is diagnosed at a later stage when chances of survival are minimum. The fear of women is obvious from the following statements.

**Code: Fear**
  P 4: “*The fear of something, like breast cancer, that is unknown to you is very difficult to face and cope with.*”
**Code: Frightening**
  P 7: “*I was very upset and frightened about my breast cancer. The very name of this disease seemed dreadful to me. I thought I would never survive and I felt so discouraged because I was healthy and fit and my eating habits were good. I felt my body and fate was disloyal to me and it seemed so unfair to me.*”

Focused education is required to increase breast health awareness in the general population of Pakistan. Men and families should be equally involved in awareness programs in order to increase openness in the male-dominant society of Pakistan.

### 3.2. Cultural Barriers

Shyness and personal modesty are common traits in all Pakistani women. All participants felt that breasts are sensitive parts of a woman’s body which have to be concealed all the time. They felt unprotected to let male practitioners do their breast examinations. They felt embarrassment in showing their breasts, even to female medical practitioners for routine tests. Issues related to breasts are not openly discussed with mothers, daughters, or other extended family members in Pakistani society. All participants confirmed that there is insufficient awareness regarding breast health among the Pakistani community which is due to lack of education and a proper forum to openly discuss these issues. These sentiments can be observed from the following statement.

P 6: “*Since childhood we are told that breasts should be kept secret. Due to lack of openness in our society, we don’t talk about it with our parents or our relatives. We only talk about it with our close friends but this discussion with our friends is not usually helpful since they too are not aware of this disease.*”

### 3.3. Early Detection is Important

Early diagnosis and treatment of breast cancer greatly increases the chances for successful cure [14]. Early detection can be achieved in two ways [3].

1. By promoting awareness about breast cancer
2. Facilitation and access to screening, which refers to the use of simple tests in order to identify breast cancer in women when they do not have visible symptoms, e.g., Mammography.

After noticing any lump or change in the breast, a doctor should be consulted immediately. In general, the more advanced cancer has grown and spread, the less is the chance that treatment will be curative [14]. In this study, all women were diagnosed early and they started treatment on time and were cured at the end, enjoying normal lives. It is true that breast cancer is a fatal disease but if it is diagnosed early and treated on time then it is not a fatal and frightening disease. The reason behind the successful treatment of all these women is the early diagnosis of their breast cancer as evident from some of the following statements.

**Code: Self Examination**
  P 7: “*After noticing a mass in my breast, I decided to find more information about it on the internet and from my friends ...Finally, I met a doctor who did a biopsy in a hospital. The biopsy revealed tumors in my breast. I was diagnosed as having Stage II breast cancer and I was told to have a surgery to be on the safe side along with chemotherapy and radiation therapy.*”
**Code: Mammogram**
  P 1: “*When I observed a change in my breasts’ size, I talked about it to my husband ...After having a mammogram which showed the existence of breast cancer in my right breast, I underwent surgery. My cancer was benign, however, my doctor recommended me to do a mammogram every six months as a precautionary measure.*”

One participant was diagnosed through her routine annual tests, one said she had started bleeding from the breast and five were diagnosed after noticing symptoms, e.g., discharge from breast, having a lump in the breast and armpit. After observing the symptoms, they consulted with doctors and after full investigations started their regular treatment. One of the seven participants has stage I breast cancer, one had stage IIIB, and five had stage II breast cancer. They all received treatment according to their stage.

### 3.4. Quality of Care and Treatment

The quality of care and treatment for breast cancer is very important for the survival of patients. Good quality and the most effective treatment programs can be regarded as those [3]

- Which are provided in a sustained and proper way
- which are linked to early detection and diagnosis
- Which stick to evidence-based standards of care.

A cancer treatment center should provide a full and complete treatment package, including guiding a patient and her family with general information and awareness, surgery, hormone therapy, chemotherapy, radiation therapy, complementary treatment and nutritional support.

All participants in the study have positive remarks about their treatment center. They all reported that the hospital staff were very cooperative and discussed everything with patients. The nurses were very friendly and the patients were given all the information they needed about their disease. The overall environment in the treatment center was very peaceful, friendly and comfortable.

**Code: Very Informative**
  P 2: “*The doctor was very informative and she spent a lot of time with me talking about the disease, the treatment, next steps and possible outcome.*”
**Code: Like Family**
  P 6: “*I was warmly received and I felt quite at home at my treatment center. Everyone was so friendly and cooperative like family.*”
**Code: Best Care**
  P 7: “*Cancer treatment requires a lot of physical, emotional, and mental courage. In this case, it is very important to have support from family and best care from your healthcare professionals, who not only focus on your treatment but also on you as a whole person.*”

All participants in this study received surgery, which is considered one of the most important procedures for breast cancer. It provides important information about the “Stage” of cancer, which is based on the size of the tumor in the breast, state of the lymph nodes and metastatic spread to other parts of the body [22].

All participants in this study received chemotherapy. Chemotherapy is used for three major purposes [23]:

1. Neo-adjuvant therapy is used to minimize or shrink cancer cells so that surgery is easier.
2. Adjuvant therapy is used to prevent or delay cancer from coming back after the initial surgery and radiation.
3. In this case, Chemotherapy is used to kill cancer cells that have spread to other parts of the body other than the breast.

Radiotherapy is a technique in which tightly controlled high energy x-rays are used after surgery to destroy any cancerous cells that may be left behind in the breast area. Six of the participants in this study received radiation therapy. If the tumor is localized and small in size then surgery or radiation alone is likely to be highly successful [3]. Chemotherapy alone can be successful for a small number of cancers, such as hematological neoplasms (leukemias and lymphomas) [3]. Hormone therapy, also called endocrine therapy, is an effective treatment for breast cancer cells that have either estrogen or progesterone receptors [24]. Hormone treatment can be used both as an adjuvant therapy (to help reduce the risk of the cancer coming back after surgery) and neoadjuvant treatment. Two out of seven participants in this study also received hormone therapy during their treatment. Choosing which techniques to use for killing cancer requires close collaboration among the entire cancer care team.

### 3.5. Support

All participants described the situation of breast cancer as very hard to face. Some thought it was a fatal disease while some became very upset to hear about breast cancer. One said, “*I want to live*”.

In these hard situations, family support gave them courage and kept them strong in facing the challenge of breast cancer and fighting the disease. Similarly, their health professional team educated them and gave them full information about the disease and gave them the courage and willpower to allow them to handle the disease.

**Code: Care Giver Support**
  P 1: “*My caregivers helped me raise my awareness about the disease and in managing the side effects. They encouraged me to have fun with wigs and some pretty, hand-knitted hats that I could wear while sleeping to keep my head warm and comfortable. I felt so blessed and grateful for their cooperation.*”
**Code: Family Support**
  P 3: “*My son, a student at the university, shaved his head and sent me a picture the night before my first chemotherapy. My son encouraged me a lot and reminded me of my strength. A lot of other family members and friends cared and cheered me on. I was not alone in this fight.*”
**Code: Husband Support**
  P 1: “*My husband, as my caregiver, persuade me to continue my treatment and helped me stay strong.*”

Thus the support of the family, friends and hospital team play an important role in making breast cancer patients strong in winning the battle.

### 3.6. Side Effects

The main side effects of chemotherapy according to participants in this study were nausea, vomiting, sleeping trouble, mood swings, aches, skin problems, weakness, sickness and hair loss.

**Code: Hair Loss**
  P 5: “*Thinking about losing my hairs was weird. It was one of the hardest moments during my treatment.*”
**Code: Side Effects**
  P 3: “*When I started my treatment and had completed my first round of chemotherapy, I noticed severe side effects. I had trouble sleeping and had severe mood swings and stomach disorders and I was not sure what would happen to me in coming days.*”

### 3.7. Courage and Willpower of Patients

Patients’ courage and will power is very important and necessary in order to win the hard and long battle against breast cancer.

**Code: Will Power**
  P 4: “*It’s very hard to know that you have breast cancer. It needs a lot of courage and willpower to do such an expensive and long treatment with possible side effects such as hair loss. But when you think about your life and your children and husband and family, you got the strength to fight.*”
**Code: Courage**
  P 5: “*My husband gave me the courage to fight. I thought how unfair this cancer is and that’s why I had to resist. I was ready to confront the fear and continue my treatment.*”

### 3.8. Learned to Face Challenges

Breast cancer is a big challenge in itself. The main treatments of breast cancer are: surgery, radiotherapy, and chemotherapy or a combination of these according to the stage in which it is diagnosed. Breast cancer treatment has many side effects including nausea, vomiting, mood swings, weakness, anxiety, sickness, and hair loss. Fighting cancer and facing the challenges of side effects is always a hard task in itself. After successfully facing these challenges and having been cured, one has obtained lessons to face any difficult situation in life.

**Code: Strength**
  P 3: “*Now that I am cured I have the strength to fight any battle. Cancer is not a good thing, but it had taught good lessons. I am now more courageous and can take things more easily.*”
**Code: Changed life**
  P 5: “*Going through the long and hard treatment of breast cancer I have learned and faced a lot. My life is totally changed now and for the betterment. I am living a healthier life and I am much more active now.*”

## 4. Limitations of the Study

This was a small study and the participants involved were fairly educated and from the main city of Peshawar. They have fairly easy access to the Internet, media and treatment centers. Their perceptions, experiences and views about breast cancers may not be equally applied to Pakistani women who live in villages and small towns and to those who are poorly educated or even illiterate.

## 5. Conclusions

More than 30% of breast cancer is diagnosed in stages III and IV in Pakistan [8]. The picture that emerged in this study is only a representative of a small segment of breast cancer patients in Pakistan, who were detected early, successfully cured and are now living a normal life. However, the insights that we gained in this study are the experiences of women in handling and managing their breast cancer from diagnosis to successful recovery. Their perceptions and set of knowledge can serve as guidelines and source of motivation for breast cancer patients, physicians, caregivers, and family members in effectively controlling and managing this curable disease in Pakistan.

Two major obstacles to achieving a successful treatment of breast cancer are: the lack of awareness and initial screening and treatment facility. This study has identified several areas to increase survival rates from breast cancer in Pakistan.

First, women in Pakistan do not know much about the curable disease of breast cancer, despite the fact that it is very common in this country. Due to lack of information, education and awareness, most women cannot answer simple questions like: what is breast cancer?; what should I do and where should I go if I have breast cancer? Comprehensive awareness and informative programs in a culturally acceptable language about this disease should be provided to all women in to minimize patient delay. Awareness and education should involve print and electronic media and informal workshops with the help of a specialist nurse and medical practitioners to provide families with concise information on breast health issues.

Second, women need easy and inexpensive access to routine examinations and mammography in order to promote early detection and minimize system delay.

Third, general practitioners need to be more educated and trained regarding the incidence and treatment of breast cancer, since these are the health professionals from whom most people in Pakistan seek medical advice initially. In particular, female staff trained to perform routine breast examinations would be very helpful in eradicating cultural barriers in early detection and management of breast cancer in Pakistan.

Finally, the importance of culture, family support, and financial concerns were highly emphasized by almost all participants in this study. Involving men and families could also lead to improved awareness and openness of society towards breast health and diseases in Pakistan.
